# Pd^0^@Polyoxometalate Nanostructures as Green Electrocatalysts: Illustrative Example of Hydrogen Production

**DOI:** 10.3390/ma3010741

**Published:** 2010-01-26

**Authors:** Rosa N. Biboum, Bineta Keita, Sylvain Franger, Charles P. Nanseu Njiki, Guangjin Zhang, Jie Zhang, Tianbo Liu, Israel-Martyr Mbomekalle, Louis Nadjo

**Affiliations:** 1 Groupe d’Electrochimie et de Photoélectrochime, Laboratoire de Chimie Physique, UMR 8000, CNRS, Université Paris-Sud 11, Bâtiment 350, 91405 Orsay Cedex, France; E-Mails: rosa.ngo-biboum-bimbong@u-psud.fr (R.N.B.); nanseu@yahoo.fr (C.P.N.N.); louis.nadjo@u-psud.fr (L.N.); 2 Laboratoire de Physico-Chimie de l'Etat Solide, ICMMO, UMR 8182, CNRS, Université Paris-Sud, Bâtiment 410, 91405 Orsay Cedex, France; E-Mail: sylvain.franger@u-psud.fr; 3 Key Laboratory of Green Process Engineering, Institute of Process Engineering, Chinese Academy of Science, 100190, Beijing, China; E-Mail: zhanggj@home.ipe.ac.cn; 4 Department of Chemistry, Lehigh University, Bethlehem, PA, 18015, USA; E-Mails: jiz306@lehigh.edu (J.Z.); liu@Lehigh.EDU (T.L.); 5 Institut Lavoisier, UMR 8180, Université de Versailles St Quentin, 45, avenue des Etats-Unis, 78035 Versailles Cedex, France; E-Mail: israel.mbomekalle@chimie.uvsq.fr

**Keywords:** green chemistry, palladium nanoparticles, nanostructures, vanadium-substituted polyoxotungstate, self-assembly, hydrogen evolution reaction

## Abstract

Green-chemistry type procedures were used to synthesize Pd^0^ nanostructures encapsulated by a vanadium-substituted Wells-Dawson-type polyoxometalate (Pd^0^@POM). The cyclic voltammogram run with the Pd^0^@POM-modified glassy carbon electrode shows well-defined waves, associated with Pd^0^ nanostructures and the V^V^/V^IV^ redox couple. The Pd^0^@POM-modified electrode displayed remarkably reproducible cyclic voltammetry patterns. The hydrogen evolution reaction (HER) was selected as an illustrative example to test the electrocatalytic behavior of the electrode. The kinetic parameters of the HER show the high efficiency of the Pd^0^@POM-modified electrode. This is the first example of electrochemical characterization of a modified electrode based on a vanado-tungstic POM and Pd^0^ nanostructures.

## 1. Introduction

Polyoxometalates (henceforth POMs for convenience), constitute a unique class of molecular metal-oxygen clusters, endowed with remarkable behaviours in several fields including catalysis, electrocatalysis, medicine and materials sciences [1,2,3,4,5]. Most POMs are anionic structures constituted of early transition-metal elements in their highest oxidation states. POMs are a versatile family of molecular metal-oxide clusters with an enormous diversity of structures. Of interest in the following, their reduced forms, owing to their electron and proton transfer and/or storage abilities, may act as efficient donors or acceptors of several electrons without structural change. Such reversible charge transfer ability makes POMs ideal candidates for electron exchange reactions. Their redox behaviours may be very flexible and finely tuned on purpose, by changing smoothly their composition. Reduced POMs have been shown to serve as reducing and capping agents for metal nanostructures. POMs are reported to adsorb on various solid materials and this property has recently been exploited for the stabilization of nanoparticles [6,7,8,9,10,11,12,13,14,15,16,17,18]. Such active forms of POMs can be generated by a variety of techniques, including electrochemistry, photochemistry and radiolysis [6,7,8]. Recently, we have shown that synthesis of POMs in which one or several addenda atoms or substituent centers are not in their highest oxidation state opens the way toward green-chemistry type procedures for the one-step synthesis and stabilization of colloidal metal nanostructures [8,9,10,11,12,13,14]. In addition of acting both as reducing and stabilizing agents, the selected POMs are operated in aqueous environment [8,9,10,11,12,13,14]. As POMs are well-characterized molecular oxides, the synthesized POM-encapsulated metal nanostructures feature well defined oxide-supported catalysts, thus opening the way for characterizing both the catalyst and the catalytic processes at a molecular level. Indeed, oxides are known to be the favourite supports for most heterogeneous catalysts.

We now report on the electrochemical characterization of the solid collected from Pd^0^@POM blackberry-containing solutions and study its ability to act as an efficient electrocatalysts. The hydrogen evolution reaction is selected as an illustrative example. Palladium is relatively abundant in the Earth’s crust, is cheaper than platinum and exhibits remarkable catalytic properties. As a consequence, palladium appears as a very promising candidate in the global search for Pt-free catalysts for various applications, including fuel cells [19,20,21,22]. However, it is important to elaborate Pd^0^ nanostructures in order to reduce the noble metal loading of catalysts. Specifically, some Pd^0^ nanostructures electrodeposited from POMs have shown remarkable behaviours in the oxygen reduction reaction [23,24] and the detection and quantification of hydrazine [25]. However, to our knowledge, electrochemical and electrocatalytic behaviors of these Pd^0^ nanostructures capped with a vanadium-substituted POM have not been reported.

## 2. Experimental

### 2.1. Electrochemistry Equipment, Apparatus and Procedures

The source, mounting and polishing of the glassy carbon (GC, Le Carbone Lorraine, France) electrodes for electrochemical studies have been described previously [26]. The electrochemical set-up was an EG & G 273 A driven by a PC with the M270 software. Potentials are quoted against a saturated calomel electrode (SCE). The counter electrode was a platinum gauze of large surface area. Pure water was used throughout. It was obtained by passing through a RiOs 8 unit followed by a Millipore-Q Academic purification set. The pH = 5 medium composition was: 0.4 M CH_3_COONa + CH_3_COOH. For lower pH values, HClO_4_ or H_2_SO_4_ were used. The solutions were deaerated thoroughly for at least 30 minutes with pure argon and kept under a positive pressure of this gas during the experiments.

### 2.2. Mother Solution Work-up

The Pd^0^@POM blackberry-containing mother solutions were centrifuged at 40,000 rpm for 60 min, and the solid collected. As the blackberries are not solid, the Pd^0^@POM formulation will be kept throughout to designate the solid collected from centrifugation of aged mother solutions. The UV-vis spectra were recorded on a Perkin Elmer Lambda 19 spectrophotometer. UV-visible spectra were also run for the supernatant solutions and found to contain little, if any, Pd^0^@POM nanostructures.

Several other complementary techniques and procedures used for characterizing the Pd^0^@POM nanostructures, have been described in detail [13] and are only briefly recalled here. The BI-ZPM laser light-scattering spectrometer (Brookhaven Instruments) equipped with a Coherent Radiation 200-mW Diode Pumped solid-state (DPSS 532) laser with a wavelength of 532 nm, and a BI-9000 correlator, was used for both SLS and DLS measurements. X-Ray photoelectron spectra (XPS) were recorded by Scienta ESCA-300 X-ray Photoelectron Spectroscopy. Transmission Electron Microscopy (TEM) was performed on a JEOL-2000FX TEM operating at an acceleration voltage of 200 kV. High resolution TEM photographs were taken on a JEOL 2200FS electron microscopy equipped with a field emission source. The operating voltage was 200 kV.

### 2.3. Electrode Preparation

A glassy carbon electrode was thoroughly polished as previously described, and then, modified as follows: a few μL of the centrifuged and washed Pd^0^@ POM nanostructures suspension in water are deposited on the polished glassy carbon surface (GC), and allowed to dry in the air at room temperature. The surface was then coated with 3 μL of 5 wt % Nafion solution for 10 µL of deposited solution and again let to dry in the air at room temperature.

## 3. Results and Discussion

### 3.1. Synthesis of HPV^IV^

Specifically, the synthesis of [H_4_V^IV^PW_17_O_62_]^9-^ (HPV^IV^) is briefly outlined. The key to preparation of pure precursor K_7_[H_4_PW_18_O_62_].18H_2_O in good yield has been described [27]. The monolacunary species, K_11_[H_4_PW_17_O_61_].18 H_2_O is then straightforwardly obtained in the presence of 1 M KHCO_3_. Finally, addition of VOSO_4_·5H_2_O acidified with concentrated HCl yields the desired K_9_[H_4_V^IV^PW_17_O_62_]·18H_2_O. Detailed preparation procedures for sixteen V-substituted POMs synthesized on purpose to illustrate their ability to generate metal nanostructures in mild conditions can be found in the relevant papers [9,27]. They all are primarily derived from α-[X_2_W_18_O_62_]^6-^ or its dissymmetrical analogue α-[H_4_XW_18_O_62_]^7-^ (X = P or As), with the possibility for some W centers to be substituted by Mo atoms in the first group. The synthesis of these sixteen POMs gives the opportunity to highlight some general trends: i) substitution of W atoms by V atoms will usually shift the pH stability domain to higher values [4]; ii) the formal potentials of Mo and V substituent centers are invariably detected in the order V^IV^/V^V^, Mo^V^/Mo^VI^ and W^V^/W^VI^ in tungstic POMs substituted in their framework both by Mo and V atoms; iii) it is possible to finely tune the formal redox potentials observed for a POM by changing its atomic composition. These compounds are stable to dioxygen and are stable over a large pH domain including pH = 7 [9,27]. It is noteworthy that these compounds can be synthesized both in the oxidized and reduced forms for the V center.

### 3.2. Pd^0^ @ POM Nanostructure Synthesis

The selected palladium salt for the synthesis was K_2_PdCl_4_ which has an apparent formal potential E^0’^(Pd^0^/[PdCl_4_]^2-^) = 0.350 V *vs.* SCE [28]. Several requirements must be fulfilled for the choice of a suitable POM for the green chemistry-type one-step synthesis of Pd^0^@ POM nanostructures [8,9,13]. Among them, availability and stability of the oxidised and the reduced forms in aerobic conditions, and also the formal potential can be cited. The well-known fully polytungstic Keggin or Dawson POMs do not meet all these requirements. Among the synthesized POMs, HPV^IV^ is the most efficient for the planned synthesis, in agreement with apparent formal potentials of its V^IV^/V^V^ redox couple (E^0^’= 0.290 V *vs.* SCE) [9,27]. Several assays prove that the synthesis follows thermodynamics.

The preparation of Pd^0^ nanoparticles by reducing [PdCl_4_]^2-^ via the Wells-Dawson-type vanadium-substituted POM, K_9_[H_4_V^IV^PW_17_O_62_], constitutes an illustrative example from which the main characteristics making this synthesis a friendly process can be highlighted [9]: the whole process is performed in aqueous media, at room temperature; the selected POM is stable from pH = 0 to pH = 7, even in the presence of oxygen; finally, this POM is stable in solution with the vanadium center in the V^IV^ or in the V^V^ form. Schematically, the stoichiometric equation of this reaction reads:

2 [H_4_V^IV^PW_17_O_62_]^9-^ + [PdCl_4_]^2-^→ 2 [H_4_V^V^PW_17_O_62_]^8-^ + Pd^0^ + 4 Cl^-^ (1)

A better formula for the species in solution would write the colloidal nanoparticles as Pd^0^ @ POM where POM represents HPV^IV^ or HPV^V^. Interestingly, this way of writing the colloid makes clear the encapsulation of the metal nanostructures by the POM and explains the negative charge of the nanoparticles preventing their agglomerization via strong electrostatic repulsion.

**Figure 1:**
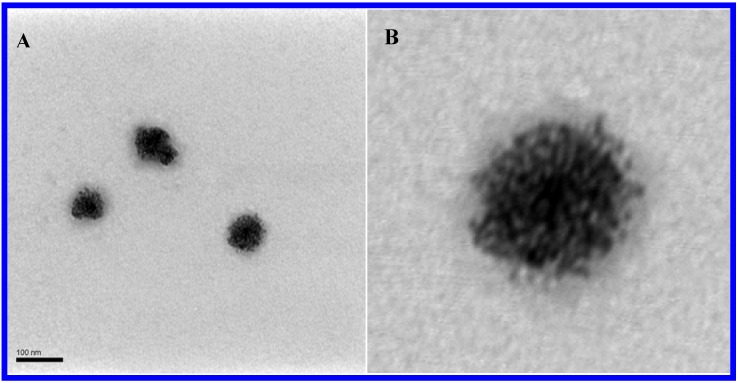
(A) Representative TEM of Pd^0^@POM blackberries and (B) an enlarged image.

The same simple procedure was used throughout, but the initial observations were slightly different in pure water and in pH = 1.5 HCl medium. Typically, 0.5 mM HPV^IV^ was coupled with K_2_PdCl_4_ in aqueous solution. Upon addition of the appropriate amount of K_2_PdCl_4_ to obtain a solution 0.9 mM in Pd salt, the formerly blue color of the POM solution immediately turns to dark brown. The synthesis was monitored by UV-vis spectroscopy. Important modifications of the initial spectrum were observed, with significant absorption increase in the longer wavelength region. Such increase, attributed to the plasmon resonance absorbance of Pd^0^ nanoparticles, eventually stopped, thus signaling the reaction completion. The solution was then worked-up. Alternatively, the pH of the synthesis medium was slightly modified to avoid hydrolysis during Pd^0^@POM blackberry preparation. Typically, 2 mM K_2_PdCl_4_ solution in water (pH = 3.54) was acidified by HCl to pH 1.5 to avoid its hydrolysis. An appropriate volume of this solution (0.05 to 0.75 mL) was mixed with 10 mL of 0.1 mM HPV^IV^ solution in water, thus providing a series of reaction mixtures with different molar ratios of K_2_PdCl_4_ to the POM HPV^IV^. The mixture was vigorously stirred for a couple of minutes before being filtered into dust-free cells through Millipore filters with 0.1 µm pore size. After a few hours of delay, the solution colour changed from blue to yellow. The reaction process was monitored by using static and dynamic light scattering techniques (SLS and (DLS), throughout the long period necessary for blackberry formation. In pH = 1.5 medium, the initially 3 nm radius hydrophilic POM-capped Pd nanoparticles are shown by laser light scattering and TEM studies to self-assemble into stable, hollow, large spherical (30–50 nm radius), single-layer “blackberry”-type structures even in dilute solutions [13], a feature also encountered with other hydrophilic macroions [29,30,31]. These spherical assemblies were shown not to be solid. TEM (Figure 1) and DLS were used to study the structure of assemblies. Complete physical characterization of these nanostructures has been carried out [13] and a detailed description is beyond the scope of the present paper.

### 3.3. Electrochemistry

**Scheme 1:**
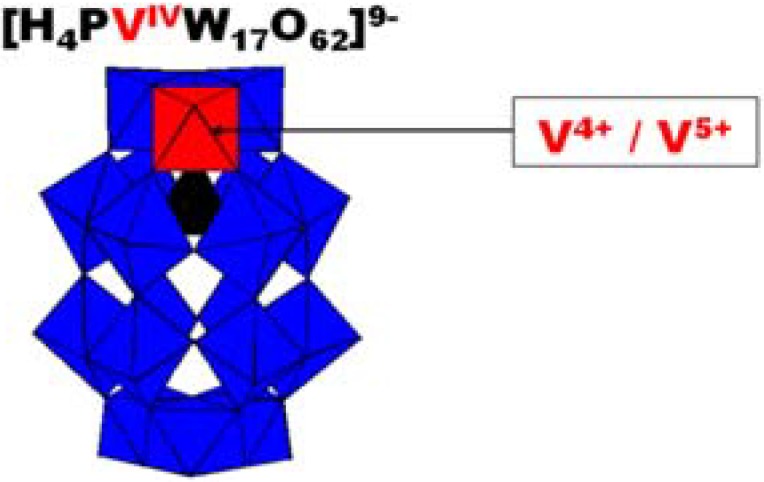
Polyhedral representation of K_9_[H_4_V^IV^PW_17_O_62_] (abbreviated as HPV^IV^) highlighting the vanadium center. For clarity, the K^+^ cations are omitted.

**Figure 2:**
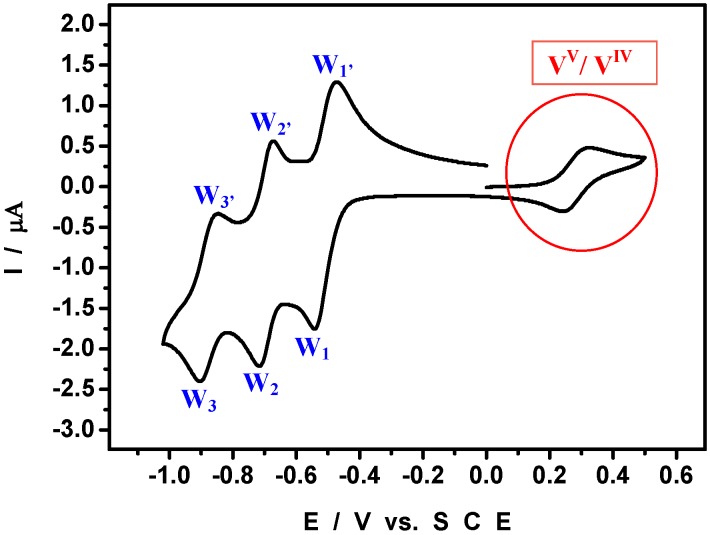
Cyclic voltammogram (CV) of 2 × 10^-4^ M HPVIV in a pH = 5 medium. The scan rate was 2 mV s^-1^; the working electrode was glassy carbon and the reference electrode was SCE.

Scheme 1 shows a polyhedral representation of HPV^IV^ in which the location of the V center is highlighted. The cyclic voltammogram (CV) run with 2 × 10^-4^ M HPV^IV^ in pH = 5 medium (Figure 2) shows that the V-wave is well-separated from those of the W framework, a feature which will aid in the specific study of the V-center. In particular, the detection of its V-center might constitute a label useful for indicating the presence of the POM or at least, of one of its elements, during the study of Pd^0^@POM nanostructures. W-waves shift roughly by 60 mV per pH unit between pH = 0 and pH = 7; in contrast, the formal potential for the V^V^/V^IV^ couple is pH-independent for pH values larger than 2.5.

As expected from XPS analysis of Pd^0^@POM nanostructures [13], the very first cyclic voltammogram run (CV), in the pH = 5 medium, with an electrode modified with Pd^0^@POM nanostructures, displays some Pd^2+^ reduction wave in addition of the Pd^0^ and vanadium redox patterns. In most samples, the corresponding wave has already disappeared upon the second voltammetric run. It is noteworthy that the modified electrodes are very stable. After several months of storage in the atmosphere of the laboratory or the acidic medium, they display the same behaviors as do freshly prepared electrodes. Alternatively, cycling in the working electrolyte for 10 h every day for more than 1month does not show any significant decay of electrode characteristics.

**Figure 3:**
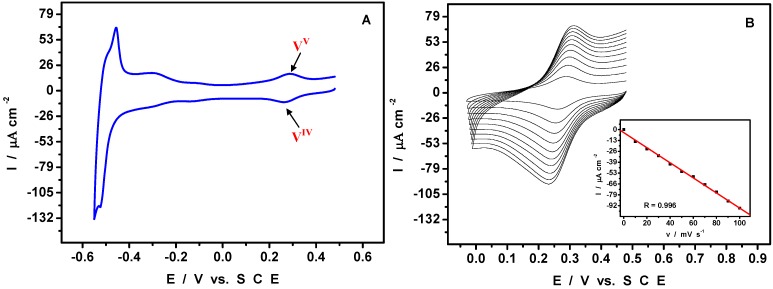
Cyclic voltammograms run in a pH = 5 medium with a Pd^0^@POM glassy carbon-modified electrode. The reference electrode was SCE.

**Figure 4:**
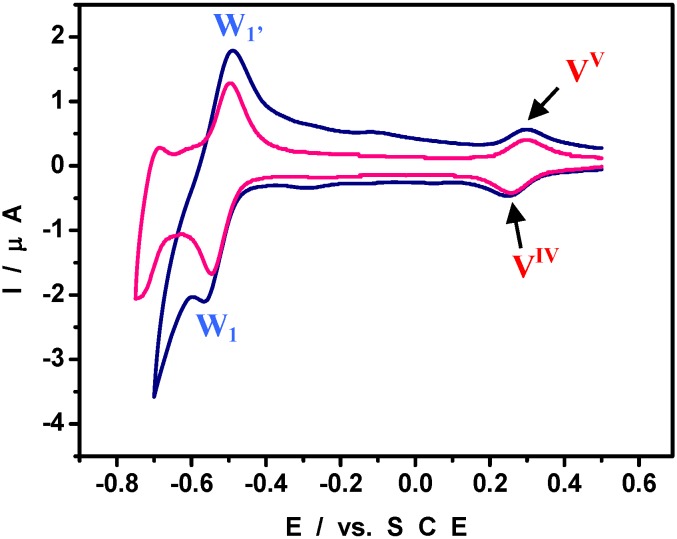
Cyclic voltammograms run in a pH = 5 medium with two differently modified glassy carbon electrodes (GC): pink curve: HPV directly adsorbed; navy curve: Pd^0^@POM film issued from the supernatant solution collected during the mother solution work-up. The reference electrode was SCE. The CV of Pd^0^@POM was scaled up to make its peak current match that observed with HPV@GC.

Figure 3A displays the CV obtained in pure pH = 5 medium. The potential domain was selected to avoid oxidation of Pd^0^ nanostructures. The redox activity of the V^V^/V^IV^ moiety is unambiguously identified by its formal potential at + 0.290 V. The pattern clearly shows that all the vanadium in Pd^0^@POM nanostructures was oxidized to the V^V^ state, in agreement with the reduction process proposed in equation (1). The good separation between the V^V^/V^IV^ redox waves from other voltammetric patterns in Figure 3A gives the opportunity for further electrochemical characterization of the V-center. Figure 3B shows the CV restricted to the V-center of HPV^V^. The reduction peak current was found to increase linearly with the potential scan rates as appears from the inset in Figure 3B. The good linearity of this variation indicates that the CVs feature a surface-controlled process. At the negative limit of the potential domain (Figure 3A), is observed the characteristic hydrogen adsorption-desorption pattern (between –0.220 V and –0.550 V) immediately followed by the hydrogen evolution itself (not shown). In agreement with its reduction peak potential location measured in Figure 2 (−0.540 V), even the first W-wave is now obscured by hydrogen adsorption processes. To add further support to this statement, the CV of the supernatant solution collected during the mother solution work-up was run. This supernatant is reminded to contain very little amount of Pd^0^@POM. Its CV turns out to clearly show the presence of both V and the first W redox couples. This chemically reversible W-wave is close to the hydrogen evolution processes which obscure the other W-waves observable in the absence of Pd nanoparticles (Figure 4). It thus appears that even this small amount of Pd^0^ is efficient enough in the electrocatalysis of the HER. Figure 4 compares the cyclic voltammogram of pre-adsorbed HPV on glassy carbon electrode and that of a film containing a very small amount of Pd^0^. The redox properties of HPV are not changed by its adsorption on glassy carbon electrode. As expected, the CV shows the characteristics of surface-controlled processes.

(A) The potential excursion was restricted to the domain where Pd^0^ electro-oxidation does not occur. The scan rate was 10 mV s^-1^. For further details, see text.

(B) The potential domain was restricted to that of the V^V^/V^IV^ redox couple, which is studied as. CVs are shown as a function of scan rate. Inset: dependence of the reduction peak current as a function of scan rate. R = 0.996.

**Figure 5:**
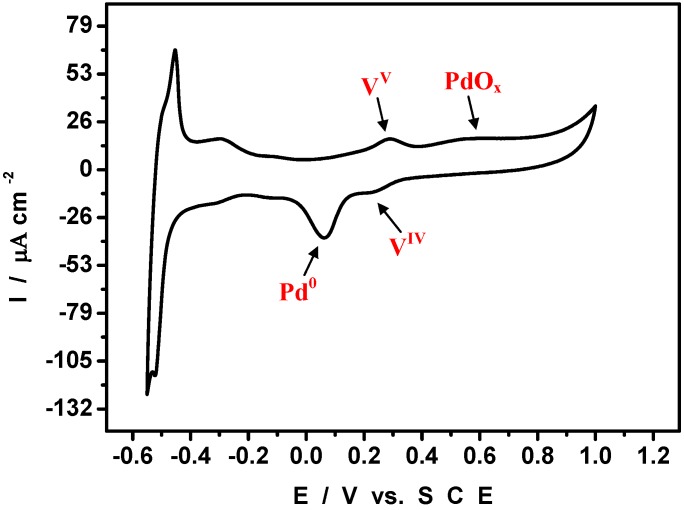
Cyclic voltammograms run in a pH = 5 medium with a Pd^0^@POM glassy carbon-modified electrode. The reference electrode was SCE. Compared to Figure 2A, the potential domain was extended toward positive values. For further details, see text.

To highlight the fingerprint of Pd^0^ nanostructures, the potential domain was extended toward more positive values than in Figure 3A. Figure 5 shows the corresponding CV. In addition to the pattern already present in Figure 3A, we observe the composite drawn-out pattern featuring the oxidation of Pd^0^ surface, prior to the oxygen evolution at the positive limit of the CV (not shown). Also the characteristic symmetrical reduction wave of palladium oxides (PdO_x_) is identified with its reduction peak located at E_p_ = + 0.08 V, clearly negative of the V^V^/V^IV^ formal potential (E^0^’= 0.290 V). A complementary experiment supports the interpretations given for Figure 3A. For example, the characteristic reduction of Pd oxides is not observed if the potential is not positive enough to oxidize Pd^0^ nanostructures. In addition, the CVs are useful for a rough estimation of the relative contents of V and Pd. The ratio was obtained by comparison of the charge of the V-wave with the charge passed in the Pd oxide stripping reaction. Several independent determinations put the charge ratios around (8 ± 1) %. Determination of the relative contents of W and Pd is unfavourable, due to pH-dependence of W-waves, and the proximity of HER processes.

**Figure 6:**
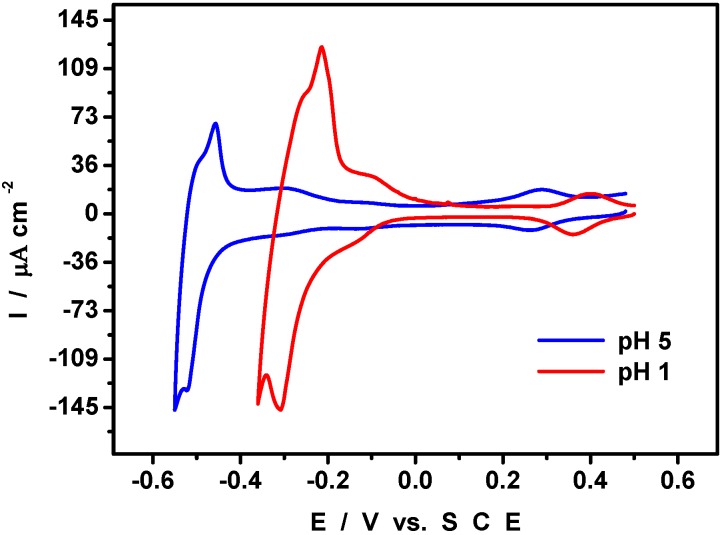
Cyclic voltammograms run at two different pH values with a Pd^0^@POM glassy carbon-modified electrode. The potential excursion was restricted to the domain where Pd^0^ electro-oxidation does not occur. The scan rate was 10 mV s^-1^; reference electrode was SCE. (red) pH = 1; (blue) pH = 5.

At least two reasons induce us to study the Pd^0^@POM-modified electrode in several pH media. First, it has previously been shown that the potential location of the V^IV^/V^V^ center depends on the solution acidity for pH values lower than 2.5. Figure 6 shows the superposition of the CVs run in pH = 1 and pH = 5 respectively. The comparison confirms a pH-dependence of the V^IV^/V^V^ redox couple between these two pH values. The characteristic features of this couple are close to those observed in the solution electrochemistry of the POM for the same pH domain. These results confirm the integrity of POMs encapsulating Pd^0^. Second, palladium oxides are known to exhibit complicated behaviors, as a function of the solution pH and of their hydration degree [32]. As a consequence, the separation between the potential locations of palladium and vanadium might not always be as favorable as observed at pH = 5. In the present work, the reduction peak potentials for palladium oxides were found at + 0.08 V, + 0.252 V and + 0.450 V respectively at pH 5, 3.3 and 1. This trend is the expected one and the shifts are quantitatively close to those previously observed with heteropoly-13-palladates [33]. Finally, we found that the difference ΔE_p_ between reduction peak potentials of palladium oxides and V-centers is large enough at pH = 5 for the peaks to be well-separated. For lower pH values, ΔE_p_ becomes smaller than 0.08 V and the two waves merge into a well-defined broad wave. Whatever the pH, the electrode can be cycled repeatedly between anodic and cathodic electrolyte limits and display reproducible patterns.

Several observations deserve emphasis. The present results add a new direct example to the now well-established presence of POMs as stabilizing agents of nanoparticles [6,7,8,9,10,11,12,13,14,15,16]. It must also be reminded that the solution containing Pd^0^@POM blackberries has been kept for more than one year and no precipitation is observed yet. A final point must also be stressed. To our knowledge, no electrochemical study of HPV^IV^ immobilized on an electrode surface is available, and it is rewarding that the V-center could be detected after the work-up of the mother solution and the deposition of the collected solid on an electrode surface. This observation underscores the robustness of HPV.

## 4. An Example of Electrocatalytic Behaviour based on Pd^0^@POM Nanostructures: Hydrogen Evolution Reaction

**Figure 7:**
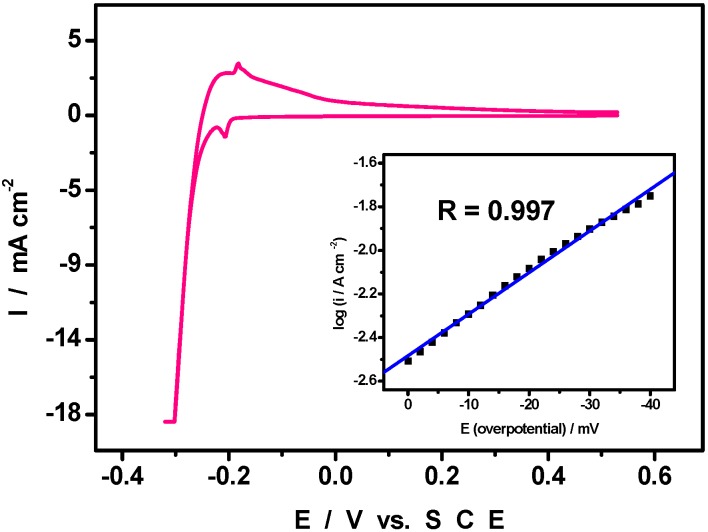
Cyclic voltammograms run in 0.5 M H_2_SO_4_ (pH = 0.30) with a Pd^0^@POM glassy carbon-modified electrode (0.2 mg Pd^0^@POM loading) and highlighting the hydrogen evolution reaction (HER). Inset: Tafel polarization curve for the HER. The scan rate was 2 mV s^-1^; reference electrode was SCE. The electrode real surface area was used.

The hydrogen evolution reaction (HER) is selected to illustrate the electrocatalytic behaviour of glassy carbon electrodes modified with Pd^0^@POM nanostructures. The POM is expected to reinforce the electrode activation and to protect the surface from poisoning. As previously described in Figure 3A, the pattern observed at the negative potential limit of the CV is attributed to the hydrogen adsorption and desorption processes just prior to hydrogen evolution. For the characterization of the HER, the fairly acidic 0.5 M H_2_SO_4_ medium was selected in order to minimize any interference of W-waves in the current to be analysed. Figure 7 shows the corresponding pattern, with a suitable current scale to highlight the hydrogen evolution itself.

During CV scans or electrolyses without stirring, bubbles are observed in the potential domain negative of the hydrogen adsorption process. Tafel-type analysis was performed on the HER wave of Figure 7 for the determination of the kinetic parameters of this reaction. In the present issue, the current density i in the Tafel equation (η = a + b logi, where η is the applied overpotential, b the Tafel slope) was expressed in ampere per unit of electrode real surface area. This electrochemically active surface area of Pd nanoparticles was estimated by integrating the charge passed in the Pd oxide stripping reaction at slow scan rates, following a literature procedure [34]. The overpotential was obtained for each current intensity of the CV of Figure 7. In addition, only the low overpotential domain was analysed. The overpotential was obtained for each current intensity of the CV of Figure 7 and a straight line was obtained through least square handling of the data. A straight line is obtained with a correlation coefficient of R = 0.997. With this data handling, the inset in Figure 7 underscores the significantly high current densities achieved with the Pd^0^@POM-modified electrode. The corresponding Tafel parameters with the exchange current density i_0_ in A cm^-2^ are logi_0_ = −3.24 to –2.48 and b = −52 mV to −83 mV. The average values (logi_0_ = −2.86 ± 0.38) are in the range obtained for the best metallic electrodes with slopes ranging from −30 to −120 mV [35], (for example, logi_0_ = −3.50 to −2.60 for Pt and −3.70 to −2.80 for Pd [36,37]). Thus the exchange current density i_0_ measured with Pd^0^@POM is 2 to 2.9 times larger than the corresponding value obtained for unmodified bulk Pd. A beneficial effect, but with a smaller enhancement, was also reported in the case of dimethylaminopyridine functionalized Pd nanoparticles [38]. The important outcome of the exchange current density measured in the present work is the confirmation that the presence of the POM has a beneficial effect on electrode activation as could be anticipated from recent work on POMs in the absence of noble metal [39]. Work is underway to study the influence of Pd^0^@POM nanostructures morphology on its HER electrocatalytic activity which is known to be extremely structure-sensitive.

## 5. Conclusions

POM-capped Pd^0^ nanostructures were prepared and self-assembled into large spherical blackberry type structures. Upon storage in the dark, without any other particular care, in the atmosphere of the laboratory, the solutions do not show precipitation for more than one year. The solid collected after centrifugation of the mother solution was used to modify a glassy carbon electrode surface. Cyclic voltammetry characterization of such electrode in different pH media, revealed that a pH = 5 buffer is suitable for a good separation of the redox characteristics of both the Pd^0^ nanostructures and the vanadium center of the POM. It is remarkable that the characteristic features of this couple are close to those observed in the solution electrochemistry of the POM. To our knowledge, this is the first solid state study of the POM used for synthesis and stabilization of palladium nanoparticles and detection of vanadium in the final solid indicates the robustness of the POM. In addition, the modified electrode strongly catalyzes the hydrogen evolution reaction, selected here as an illustrative example. The electrode is also robust and durable and could be kept for several months in the atmosphere of the laboratory. Overall, this work also amounts to a study of aging of the catalyst constituted by the POM encapsulated Pd^0^ nanostructures, Pd^0^@POM. This first example demonstrates the long term stability and the efficiency of this electrocatalyst. Hopefully, other successful electrocatalytic processes could be triggered by the electrode. Future work will examine the possibility to use high surface area appropriate conducting supports for such catalysts. Particular attention will be paid to possible synergistic effects between the two partners of this catalyst.
